# COMP report: CPQR technical quality control guidelines for Gamma Knife radiosurgery

**DOI:** 10.1002/acm2.12406

**Published:** 2018-07-22

**Authors:** Anita Berndt, Monique van Prooijen, Mathieu Guillot

**Affiliations:** ^1^ Department of Medical Physics CancerCare Manitoba Winnipeg MB Canada; ^2^ Department of Medical Physics Princess Margaret Cancer Centre University Health Network Toronto ON Canada; ^3^ Département de Radio‐Oncologie Centre hospitalier universitaire de Sherbrooke Sherbrooke QC Canada

**Keywords:** Gamma Knife, quality assurance, radiosurgery

## Abstract

The Canadian Organization of Medical Physicists (COMP), in close partnership with the Canadian Partnership for Quality Radiotherapy (CPQR), has developed a series of Technical Quality Control (TQC) guidelines for radiation treatment equipment. These guidelines outline the performance objectives that equipment should meet in order to ensure an acceptable level of radiation treatment quality. The TQC guidelines have been rigorously reviewed and field tested in a variety of Canadian radiation treatment facilities. The development process enables rapid review and update to keep the guidelines current with changes in technology (the most updated version of this guideline can be found on the CPQR website). This particular TQC details recommended quality control testing for Gamma Knife radiosurgery.

## 1 INTRODUCTION

The CPQR is an alliance among the three key national professional organizations involved in the delivery of radiation treatment in Canada: the Canadian Association of Radiation Oncology (CARO), the Canadian Organization of Medical Physicists (COMP), and the Canadian Association of Medical Radiation Technologists (CAMRT). Financial and strategic backing is provided by the federal government through the Canadian Partnership Against Cancer (CPAC), a national resource for advancing cancer prevention and treatment. The mandate of the CPQR is to support the universal availability of high quality and safe radiotherapy for all Canadians through system performance improvement and the development of consensus‐based guidelines and indicators to aid in radiation treatment program development and evaluation.

This publication, *CQPR TQC Guidelines for Gamma Knife Radiosurgery*, contains detailed performance objectives and safety criteria for *Gamma Knife Radiosurgery*. The development of the individual Technical Quality Control (TQC) guidelines is spearheaded by expert reviewers and involves broad stakeholder input from the medical physics and radiation oncology community.^1^ The development process enables rapid review and update to keep the guidelines current with changes in technology (the most updated version of this guideline can be found on the Canadian Partnership for Quality Radiotherapy (CPQR) website). This article details recommended quality control testing for Gamma Knife radiosurgery. Please refer to the overarching document *TQC Guidelines for Canadian Radiation Treatment Centres* 2 for a programmatic overview of technical quality control, and a description of how the performance objectives and criteria listed in this document should be interpreted.

All information contained in this document is intended to be used at the discretion of each individual center to help guide quality and safety program improvement. There are no legal standards supporting this document; specific federal or provincial regulations and license conditions take precedence over the content of this document.

## 2 SYSTEM DESCRIPTION

The Gamma Knife (GK) Perfexion™ (Elekta AB, Stockholm, Sweden) is used to treat intracranial lesions using stereotactic radiosurgery (SRS) procedures. Radiation is delivered by means of 192 ^60^Co sources arranged in rings with a common focus point.^3^, ^4^ By distributing the incident radiation over nearly the entire brain, a very large dose can be delivered to a well localized target with minimal harm to healthy brain tissue. These single fraction treatments are a less invasive alternative to cranial surgery.

The 1 mm × 20 mm ^60^Co sources are encapsulated in bushings and are arranged in eight sectors. The sectors move independently to position a subset of 24 sources over one of three different hole sizes in a tungsten collimator or to an “off” (blocked) position between holes. This allows the delivery of “marbles” of radiation (4 mm, 8 mm, or 16 mm shots), which can be combined to conform to the shape of the tumor. Since the different field sizes are created by means of precisely machined collimators, and the radiation is delivered using ^60^Co, much of the variability in dose delivery associated with other external beam devices is eliminated.

Dose rates at the center of an 8 cm radius polystyrene sphere are on the order of 3.5 Gy/min for a newly loaded unit. The sources are enclosed within an iron “ball”; additional shielding for scattered radiation is provided by sliding shutters. No primary radiation exits the unit.

Before imaging, a frame is fixed to the head of the patient. This serves two purposes: to define a coordinate system common to the imaging, planning, and treatment system, and to ensure that the patient cannot move during treatment. The patient positioning system (PPS; treatment couch) is rigidly affixed to the treatment unit, and the head frame is in turn locked into place on the PPS. Drive motors within the couch automatically position the patient to the prescribed isocenters during treatment. Head frame immobilization and high mechanical reproducibility allow for the accuracy required to deliver large doses to targets near relevant structures within the brain.

## 3 RELATED TECHNICAL QUALITY CONTROL GUIDELINES

In order to comprehensively assess GK performance, additional guideline tests, as outlined in related CPQR TQC guidelines must also be completed and documented, as applicable. Related TQC guidelines, available at cpqr.ca, include:

- Safety Systems
- Major Dosimetry Equipment

## 4 TEST TABLES

Tables 1, 2, 3 list the daily/weekly, monthly/quarterly and annual recommended quality control tests.

**Table 1:** Daily/weekly quality control tests

Designator	Test	Performance	
		Tolerance	Action
Daily			
D1	GK unit interlocks (frame adapter, side panels)	Functional	
D2	Timer accuracy, linearity	1%, 0.5%	2%, 1%
D3	Treatment console alarm test	Functional	
D4	Emergency procedure placards	Present	
Weekly			
W1	Focus precision test	Functional	

**Table 2:** Monthly/quarterly quality control tests

Designator	Test	Performance	
		Tolerance	Action
Monthly			
M1	Clearance test tool check	Functional	
M2	UPS battery check	Functional	
M3	Patient positioning system retraction	Functional	
M4	Patient positioning system accuracy	n/a	0.5 mm
Quarterly			
Q1	Sector alignment	n/a	0.5/1.0 mm^a^

a 0.5 mm for 4 and 8 mm collimators, 1.0 mm for 16 mm collimator.

**Table 3:** Annual quality control tests

Designator	Test	Performance	
		Tolerance	Action
Annual			
A1	Coincidence of radiation and mechanical isocenter	0.1 mm relative to baseline	0.5 mm absolute
A2	Timer linearity	0.5%	1%
A3	Timer transit error	Baseline	
A4	Profile accuracy	n/a	1 mm
A5	Backup timer on GK sector computer	Functional	
A6	Absolute calibration	1%	2%
A7	External service dose verification	n/a	5%^a^
A8	End‐to‐end test	1–5%/0.5 mm	1–5%/1.0 mm
A9	Radiation leak test	Baseline	
A10	Radiation survey	Background	
A11	Independent quality control review	Complete	

a After each major maintenance and then every other year; tolerance as per testing institution (e.g., Imaging and Radiation Oncology Core [IROC]).

### Notes for Daily/Weekly Tests

**Table:** 

D1	The GK inhibits beam on if the patient is not locked in place, at the correct gamma angle with the side protection panels engaged
D2	The GK timer agrees with an independent measurement (e.g., stopwatch). Linearity can be tested by cycling through shots of different durations over multiple days
D3	The GK built‐in alarm test causes the console alarm to sound
D4	The emergency procedure placards are posted
W1	The GK built‐in focus precision test indicates “PASS”

### Notes for Monthly/Quarterly Tests

**Table:** 

M1	The GK clearance test tool check passes. Also check after possible damage to the tool. At the discretion of the physicist, test frequency may be reduced to semiannually
M2	The Elekta uninterruptible power supply (UPS) check passes
M3	Disengaging the x/z couch clutch allows the couch to be manually moved in the x/z direction
M4	The position of the patient positioning system must be verified against physical reference positions over an appropriate clinical range in the directions of the three axes (x,y,z)
Q1	The sectors move to correct alignment with the 4, 8, or 16 mm collimators

### Notes for Annual Tests

**Table:** 

A1	The positions of the radiation and mechanical isocenters agree with each other
A2	The GK timer is linear. Test over a larger range than daily testing
A3	The transit error is consistent with that measured during commissioning
A4	The measured profiles agree with those in the treatment planning system. Stated tolerance applies to the 50% isodose line for each collimator size
A5	The backup timer on the GK sector computer agrees with the console computer
A6	The absolute dose rate in the treatment planning system matches the measured dose rate. Measurements must be made with a calibrated chamber using an accepted protocol (e.g., TG‐21^5^)
A7	The absolute dose is independently verified by an external service (e.g., IROC Houston OSLD/TLD [optically‐stimulated/thermoluminescent dosimeter] Monitoring Program)
A8	An end‐to‐end phantom test is performed including frame placement, imaging, treatment planning, treatment, and verification that the intended treatment was delivered with the stated dose and positioning accuracy. The dosimetric accuracy depends on the dosimeter being used. For example, 1% accuracy would apply when using an ion chamber, whereas 5% would be appropriate for film
A9–10	The configuration of these tests will depend on the design of the facility and equipment. As a minimum, Canadian Nuclear Safety Commission (CNSC) license conditions and applicable regulations must be followed
A11	To ensure redundancy and adequate monitoring, a second qualified medical physicist must independently verify the implementation, analysis, and interpretation of the quality control tests at least annually

## CONFLICT OF INTEREST

The authors have no other relevant conflicts of interest to disclose.
